# Machine learning instructed microfluidic synthesis of curcumin-loaded liposomes

**DOI:** 10.1007/s10544-023-00671-1

**Published:** 2023-08-05

**Authors:** Valentina Di Francesco, Daniela P. Boso, Thomas L. Moore, Bernhard A. Schrefler, Paolo Decuzzi

**Affiliations:** 1 Laboratory of Nanotechnology for Precision Medicine, Istituto Italiano Di Tecnologia, Via Morego 30, Genova, 16163 Italy; 2 Department of Civil, Environmental and Architectural Engineering, University of Padova, Via Marzolo 9, Padova, 35131 Italy; 3 Institute for Advanced Studies, Technical University of Munich, Lichtenbergstraße 2 a, 85748 Garching, Germany

**Keywords:** Nanomedicine, Drug delivery, Microfluidics, Artificial neural network, Artificial Intelligence

## Abstract

The association of machine learning (ML) tools with the synthesis of nanoparticles has the potential to streamline the development of more efficient and effective nanomedicines. The continuous-flow synthesis of nanoparticles via microfluidics represents an ideal playground for ML tools, where multiple engineering parameters – flow rates and mixing configurations, type and concentrations of the reagents – contribute in a non-trivial fashion to determine the resultant morphological and pharmacological attributes of nanomedicines. Here we present the application of ML models towards the microfluidic-based synthesis of liposomes loaded with a model hydrophobic therapeutic agent, curcumin. After generating over 200 different liposome configurations by systematically modulating flow rates, lipid concentrations, organic:water mixing volume ratios, support-vector machine models and feed-forward artificial neural networks were trained to predict, respectively, the liposome dispersity/stability and size. This work presents an initial step towards the application and cultivation of ML models to instruct the microfluidic formulation of nanoparticles.

**Supplementary Information:**

The online version contains supplementary material available at 10.1007/s10544-023-00671-1.

## Introduction

The rapid development and deployment of the mRNA lipid nanoparticle vaccines against the SARS-CoV-2 virus brought to the forefront the potential of nanomedicine in tackling our most pressing medical challenges (Cohen 2021; Hou et al. 2021). However, conventional laboratory preparation techniques for nanoparticles (i.e. batchwise production) are not easily scaled to industrial levels. Therefore, from a translational perspective, continuous production processes such as microfluidic nanoparticle synthesis offer an attractive and scalable means to generate highly reproducible nanoparticles (Shepherd et al. 2021). In microfluidic synthesis, nanoparticle precursor materials are dispersed in either aqueous or organic solvents, and by passing these solutions through a channel with micrometer dimension with different geometries (e.g. T-junction, Y-shaped, etc.) or with different channel paths, rapid mixing can result in phenomena conducive to self-assembly of nanoparticles (e.g. micro-mixing, nano-droplet formation, or nanoprecipitation). Microfluidics offers precise control over multiple synthesis parameters (e.g. flow rate, solvent ratios, precursor concentrations) and has been shown to yield particles with better control over size as well as dispersity compared to batchwise synthesis methods (Karnik et al. 2008). Moreover, this approach can yield many different types of particles, such as liposomes (Sedighi et al. 2019), polymeric nanoparticles (Karnik et al. 2008), and hybrid solid-lipid nanoparticles (Arduino et al. 2021). In microfluidic nanoparticle development, the optimization of the desired particle attributes (size, stability, therapeutic loading, etc.) can be challenging due to the number of possible combinations of the engineering parameters. Identifying the optimal synthesis parameters is a non-trivial, time-consuming, laborious, and resource-intensive experimental task. However, advanced computational methods can help reduce the experimental burden and accelerate the identification of a ‘lead configuration’ for the nanomedicine.

The application of artificial intelligence (AI) methods to the “wet sciences” has the potential to revolutionize the way we design and optimize formulation development. AI has been applied towards numerous areas of nanomedicine, such as formulation development (Bannigan et al. 2021), microfluidics (Galan et al. 2020; Liu et al. 2021), optimizing drug delivery (Hassanzadeh et al. 2019), and better understanding nanotoxicology (Singh et al. 2020) and nanoparticle-cell interactions (Boehnke et al. 2022; Hassan 2020; Price and Gesquiere 2019). Computer algorithms and computational models that can predict the physico-chemical properties of nanosystems could streamline the development of nanoparticle systems, reducing development time as well as resource waste, resulting in faster production of reliable nanoparticle platforms for drug delivery (Boso et al. 2020; Boso et al. 2011; Yamankurt et al. 2019). Here, we report the implementation of two different machine learning (ML) tools that can aid in predicting the dispersity, stability, and size of liposomes formulated through a microfluidic device. The rationale for choosing these particular endpoints was the well-known effect that nanomedicine physico-chemical properties (i.e. size, dispersity, surface charge, shape) have on nanoparticle safety and toxicity (Fischer and Chan 2007), as well as in mediating particle interactions with biological systems (e.g. cells, biodistribution and pharmacokinetics) (Chithrani et al. 2006; Danaei et al. 2018; Di Francesco et al. 2021; Kinnear et al. 2017; Nel et al. 2009).

Liposomes were selected as a model nanoparticle system, though the methods herein are extensible to other nanoparticle drug delivery systems and microfluidic platforms. A library of liposome formulations obtained by varying the total flow rate, aqueous:organic mixing volume ratio, organic phase concentration, and curcumin loading, was systematically synthesized via a benchtop, commercially available microfluidic-based instrument. From this library, two supervised ML models were trained. One model exploits the support-vector machines (SVM) algorithm using open-source tools, and could predict particle dispersity (i.e. monodisperse vs. polydisperse) as well as particle stability (i.e. remaining monodisperse for at least 3-days). The other model is based on feed-forward artificial neural networks (ANN) and could give a quantitative prediction of the resulting liposome diameter from the initial microfluidic parameters. This work represents a step towards building a platform technology which could be widely deployed by researchers in the nanomedicine/drug delivery field towards increasing the speed at which new nanoparticle formulations can be developed.

## Results & discussion

### Generating and characterizing a library of liposomes

**Fig. 1:**
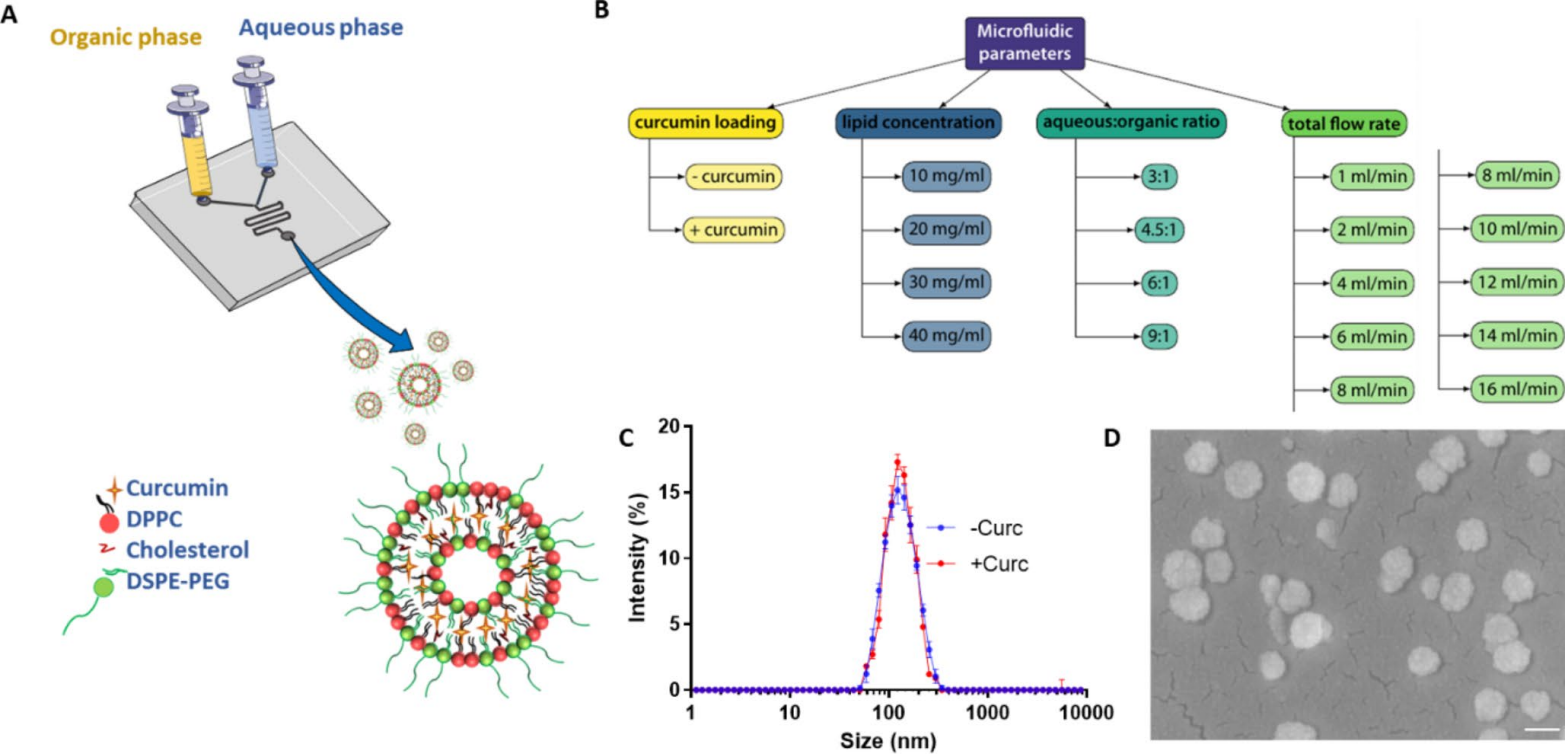
Microfluidic-based fabrication of curcumin-loaded liposomes. (**A**) Schematic representation of the microfluidic chip with the reagents dispersed in the organic (yellow) and aqueous (azure) phases used for the liposome synthesis; (**B**) List of the four independent engineering parameters (total lipid concentration; aqueous: organic volume ratio; total flow rate (TFR); drug loading) for the liposome synthesis; (**C**) Dynamic light scattering characterization of a representative liposome configuration with (+ Curc: red line) and without (- Curc: blue line) curcumin; (**D**) Scanning electron microscopy image of a representative liposome configuration (scale bar: 100 nm)

The library of liposomes were synthesized on a commercially available benchtop microfluidic system (NanoAssemblr®) comprised of two inlets for the organic and aqueous phases, a disposable cartridge with a microfluidic channel, within which the two phases are vigorously mixed, and an outlet for collecting the resulting suspension of liposomes (Fig. 1a). The aqueous phase contained phosphate buffered saline (PBS) and the organic phase contained dipalmitoyl-phosphatidyl-choline (DPPC), cholesterol (Chol), 1,2-distearoyl-sn-glycero-3-phosphoethanolamine-N-[carboxy(polyethylene-glycol)-2000] (DSPE-PEG2000), and curcumin (Curc) dissolved in ethanol. Four engineering parameters were systematically changed to realize over 200 different liposome configurations (Fig. 1b): the total flow rate (TFR), defined as the sum of the flow rates for the aqueous and organic phases, ranging between 1 and 16 ml/min; the total lipid concentration including DPPC, Chol, and DSPE-PEG2000 initially dispersed in the organic phase, varying from 10 to 40 mg/ml; the volume ratio between the aqueous and organic phases, ranging from 3 to 9; and finally the addition or omission of curcumin as a model therapeutic. Figure 1c,d present a dynamic light scattering (DLS) size distribution and a scanning electron microscopy image of a representative liposome configurations reporting on the dispersity and overall spherical shape of the obtained lipid particles.

All the liposome formulations were characterized by dynamic light scattering (DLS) to evaluate their hydrodynamic diameter (i.e. size) and polydispersity index (PdI). Furthermore, the colloidal stability of the liposome was assessed by performing DLS measurements longitudinally in time up to 72 h for liposomes stored in PBS at 37 °C. Particle size distribution (i.e. dispersity) was evaluated in context of the PdI, a dimensionless number providing quantitative information on the homogeneity of the nanoparticle population. It is here important to highlight that the definition of monodisperse (i.e. a homogenous size distribution) and polydisperse (i.e. a heterogenous size distribution) varies. Some classify monodisperse particles as having a PdI < 0.07 (Wren et al. 2020) or a PdI < 0.3 (Danaei et al. 2018), while others define PdI < 0.1 as highly monodisperse, a PdI from 0.1 to 0.4 as moderately polydisperse, and a PdI > 0.4 as highly polydisperse (Bhattacharjee 2016). Thus, although the strict interpretation of “monodispersity” should be a PdI < 0.07, most nanomedicines are considered monodisperse if the PdI is generally lower than 0.3 (Baalousha and Lead 2013). In fact, the definition of monodispersity depends to a point on the proposed application, in that a polydisperse particle size population will behave characteristically different than a monodisperse one. Moreover, FDA guidelines for liposomal formulations do not define a strict definition for monodispersity, and rather detail that adequate characterization of the liposome physicochemical properties are required (FDA 2022; Tinkle et al. 2014; Wang and Grainger 2022). Here, we define monodisperse formulations as those with a PdI ≤ 0.220 (i.e. 0.200 + 10%), and also further evaluate multiple classifications (i.e. PdI ≤ 0.200, PdI ≤ 0.300, and polydisperse). Moreover, liposomes were defined as stable if a sufficiently low PDI (≤ 0.220) was maintained over time (i.e. up to 72 h).

**Fig. 2:**
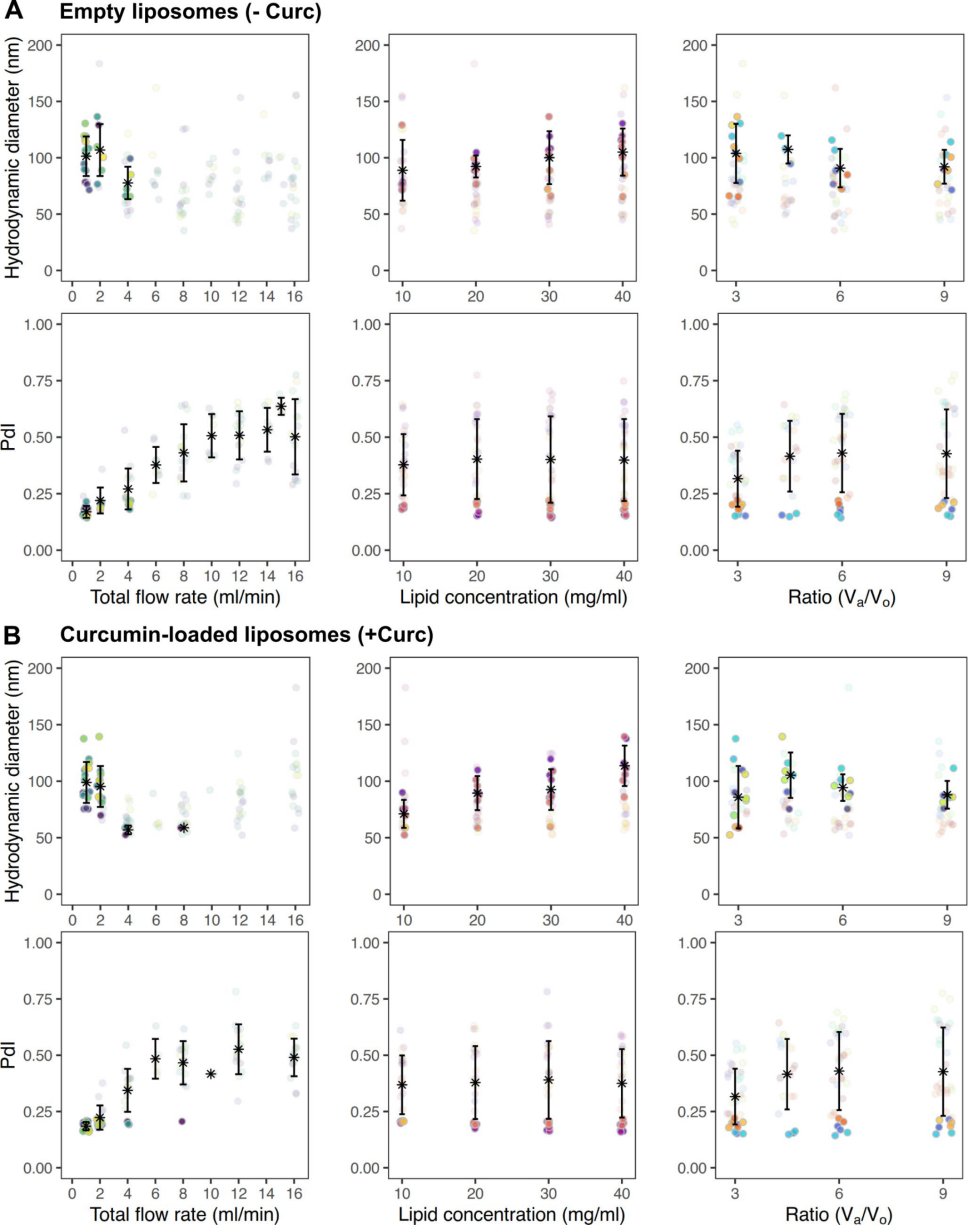
Summarized hydrodynamic diameter and PDI for all liposomal configurations. Average diameter and PdI values were calculated for each unique formulation and separated by (**A**) empty liposomes and (**B**) curcumin-loaded liposomes. Solid points represent monodisperse formulations (PdI ≤ 0.220) and translucent points represent polydisperse formulations (PdI > 0.220). Each column fixes a unique parameter as the independent variable (e.g. flow rate, lipid concentration, or aqueous:organic ratio), and for each column the different colors indicate unique formulations (i.e. unique combinations of the other two non-independent variable factors). Asterisk and error bars represent mean values ± standard deviation. For diameter averages, only monodisperse formulations were considered while for PdI averages all measurements were considered

Figure 2 reports the mean hydrodynamic diameter and polydispersity index for all 218 different liposomal configurations in terms of the four engineering parameters – total flow rate (first column); total lipid concentration (second column); aqueous:organic phase volume ratio V_a_/V_o_ (third column); and inclusion of curcumin (fourth column). For each distinct engineering parameter (i.e. column), the colored dots represent unique combinations of the other three parameters. From this scatter plot some general trends can be deduced as, for instance, an increase in the total flow rate results in a reduction of particle size, as well as an increase in PdI. Furthermore, increasing the lipid concentration seems to indicate a general increase in particle diameter, while increasing the V_a_/V_o_ leads to a narrowing of the range of particle sizes produced. However, it is difficult to extract the independent contribution of each of these engineering parameters. Interestingly, when looking at the summarized data, it was found that there were more monodisperse formulations with curcumin-loaded liposomes (31% of formulations) compared to empty liposomes (17% of formulations). For empty liposomes the sizes ranged from 35 to 183 nm, while for curcumin-loaded liposomes it was similarly 52 to 183 nm. However, for both empty and curcumin-loaded formulations the mean diameter for all formulations was around 83 ± 25 nm.

**Fig. 3:**
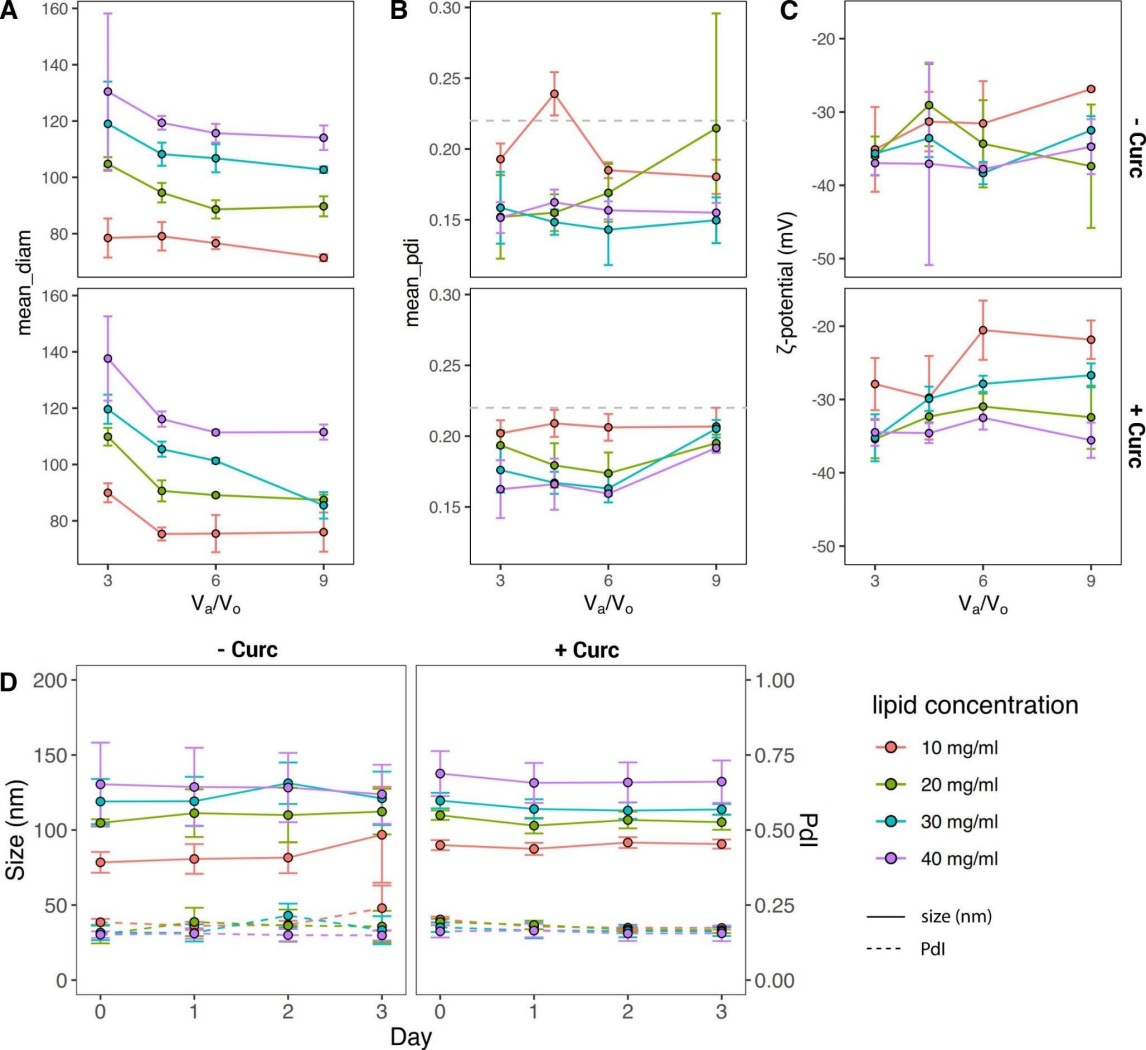
Morphological and surface characterizations of liposomes (TFR = 1 ml/min). (A) Hydrodynamic diameter, (**B**) polydispersity index (PdI), and (**C**) surface ζ-potential of liposomes as a function of the aqueous:organic volume ratio V_a_/V_o_ and for different total lipid concentrations (10, 20, 30, and 40 mg/mL). Top row is for empty liposomes (- Curc), bottom row is for curcumin-loaded liposomes (+ Curc). (**D**) Colloidal stability documented by measuring the hydrodynamic diameter and PDI over 3 days of liposomes realized at TFR 1 ml/min and V_a_/V_o_ = 3.

Traditionally, to further explore these data one would fix a variable and then analyze the contribution of the remaining parameters. Figure 3 shows, for example, the total flow rate fixed to 1 ml/min, while the other three engineering parameters vary within the predetermined ranges. In particular, Fig. 3a documents that an increase of the total lipid concentration from 10 to 40 mg/ml leads to a consistent increase in particle size from about 90 to 140 nm, whereas an increase of the aqueous to organic volume ratio V_a_/V_o_ from 3 to 9 is responsible for a decrease in size of about 20% for all considered lipid concentrations. Importantly, at this low TFR, all liposome configurations presented a PdI < 0.220 and could be therefore considered as monodisperse liposomal configurations (Fig. 3b). The surface ζ-potential of the liposomes did not change significantly with the total lipid mass, aqueous to organic volume ratios V_a_/V_o_, and addition of curcumin, ranging between − 40 and − 20 mV (Fig. 3c). This would indicate that the surface properties of the liposomal configurations are preserved despite the different microfluidic fabrication conditions. The colloidal stability was assessed for all formulations over 72 h. In particular, Fig. 3d shows the stability for the liposomes synthetized at TFR of 1 ml/min and for V_a_/V_o_ = 3. Under these conditions, the liposomes are very stable exhibiting a modest change in size and PdI of ~ 10% for the entire observation period. These same characterizations were performed for all formulations (Supporting Figure S1-2). In general, liposomes realized with higher lipid concentrations and lower TFR tended to be monodisperse and stable.

### Machine learning algorithms

ML is a branch of AI whereby a machine (computer or program) can learn without being explicitly programmed through iteratively training on historical data. Using machine learning algorithms, computational models can be built which can find patterns and trends in large data sets, which would not be obvious to the human eye. The initial library of liposome measurements was extensive: 3,518 total measurements. At the outset of the project, 2x curcumin states were tested (- Curc or + Curc), 4x lipid concentrations (10, 20, 30 and 40 mg/ml), 5x initial TFR (1, 4, 8, 12, and 16 ml/min), and 3x V_a_/V_o_ (3, 6, 9). However, throughout the building and testing of the various models, new conditions were added, including new TFR values (2, 6, 10, 14, 15 ml/min) and the V_a_/V_o_ ratio of 4.5. These new conditions were less systematically explored and more used to test the current iterations of the models on unseen data and conditions. The resulting measurements were subsequently added to the entire data set. Likewise, while testing model responses some formulations were replicated up to n = 15 times. However, for all finalized models the total number of replicates was limited to a maximum of n = 6, as described in the Materials & Methods section. All of these measurements resulted in 218 unique formulations. Of these 218 unique formulations, 72 (33%) were found to be monodisperse (PdI ≤ 0.220) directly after synthesis. A summary of measurements can be found in Supporting Table S1.

**Table 1:** Predicted model accuracy of various classification algorithms for liposome dispersity. Accuracy predicted using *k*-fold cross-validation (*k* = 10 folds) for various classification algorithms (LOR: logistic regression, LDA: linear discriminant analysis, KNN: *k*-nearest neighbor, CART: classification and regression trees, GNB: Gaussian Naive Bayes, SVM: support-vector machines). All the data are presented as mean % ± SD. (^§^ monodisperse (PdI ≤ 0.220) or polydisperse)

Classification model	Predicted accuracy for binary classification^§^	Predicted accuracy for multiple dispersity classifications
LOR	90.2 ± 3.1	74.8 ± 4.1
LDA	88.7 ± 3.9	71.2 ± 3.5
KNN	89.1 ± 4.1	74.2 ± 5.0
CART	92.3 ± 3.4	76.8 ± 3.5
GNB	87.2 ± 3.5	71.2 ± 3.2
SVM	91.3 ± 3.9	78.3 ± 5.9

#### Open-source tools for supervised machine learning models

The development of open-source tools, such as the *scikit-learn* module in Python (Pedregosa et al. 2011), offers a chance to apply powerful machine learning algorithms towards interesting and critical research questions. Here, classification-based supervised machine learning models were developed to perform qualitative predictions on liposomal dispersity and stability over time. DLS measurements were used to train ML models on the four independent engineering parameters. For training the classification models, the DLS measurements were randomly split into a training data set (85% of the observations) and a data set used to validate the ML model (15% of observations), following an approach known as holdout with random resampling (Diamantidis et al. 2000). Different classification algorithms (e.g. logistic regression, linear discriminant analysis, *k*-nearest neighbor, classification and regression trees, Gaussian Naive Bayes, and support-vector machines) were trained on the data set to build predictive models for liposomal dispersity or stability. For all the classification models, a stratified *k*-fold cross-validation was performed to estimate the model accuracy (Diamantidis et al. 2000). In this approach, the data set was split into a number of “folds” (i.e. k = 10 folds), where the class (i.e. monodisperse vs. polydisperse or stable vs. unstable) mean and variance of each fold approximated that of the whole data set. The algorithm was then run *k* times and, on the *k* = *i-*th run, the *i-*th fold was withheld as the validation data set. This approach enabled an estimation of model accuracy with smaller bias compared to the holdout with random resampling method. Table 1 shows the predicted accuracy from the stratified k-fold cross-validation and the measured accuracy of the different classification models in assessing liposome dispersity. From these results, the support-vector machines (SVM) model (Gunn 1998) was chosen for predicting particle dispersity, as it was found to be quite accurate (94.9%) using the holdout with random resampling method, while also returning among the highest predicted accuracies (91.3 ± 3.9%) using the stratified *k*-fold cross-validation. Moreover, this model was most accurate when predicting liposome stability.

**Fig. 4:**
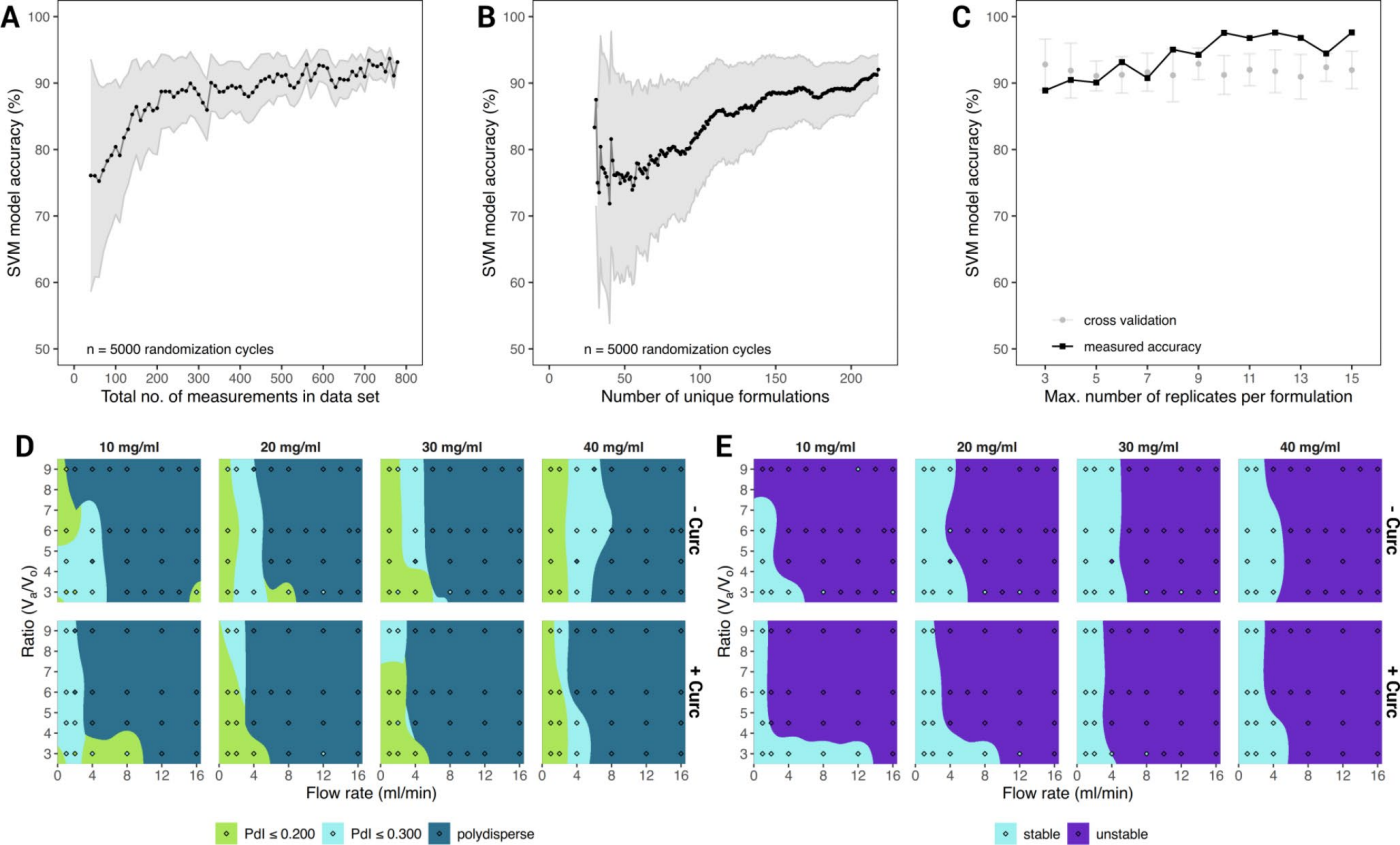
Support-vector machines (SVM) training to predict liposome dispersity and stability. (A) The effect of the liposome library size and number of experimental replicates on the model accuracy; (**B**) The effect of the number of liposome configurations on the model accuracy. (Dots represent mean values and gray areas represent mean ± 1 standard deviation); (**C**) The effect of the number of experimental replicates per formulation on SVM model accuracy. (Black dots represent the measured accuracy of the SVM model. Gray dots represent the mean model accuracy ± 1 standard deviation following a stratified *k*-fold cross-validation (*k* = 10); D. and E. Heatmaps presenting the predictions for the liposome dispersity and liposome stability, post SVM training ( filled diamonds corresponds to the actual experimental values)

In order to evaluate the effect of data set size (i.e. population) and the influence of the number of unique formulations on predicting particle dispersity, a program was written that would randomly remove measurements from the data set so that the total number of measurements would range from 40 to 779 (i.e. the maximum number of “Day 0” measurements made – Supporting Table S1). The complete supervised learning process was then re-run on this random data set, that is to say splitting into training and validation data sets, training the SVM model, and evaluating model accuracy on the validation data set. Since this process was biased by which measurements were randomly removed, the randomization procedure was repeated 5,000 times and the prediction outcomes were averaged. As expected, the variability due to random sampling (light gray shaded area in Fig. 4a) was inversely related to the total number of measurements, and the model accuracy plateaued around 550 measurements, as determined by a one-sided T-test comparing the number of measurements to a fixed model accuracy measured at 92%. During this randomization procedure, the program also counted the number of unique formulations. These data were then plotted against the SVM model accuracy in Fig. 4b. Here, similarly, the variability decreased as the number of unique formulations increased, however a clear plateaued was not identified within the limit of the current analysis (218 unique configurations).

During the experimental characterization, all the liposomal configurations were replicated a minimum of three times. However, for some configurations, the number of replicates was increased up to 15. Nevertheless, increasing the number of replications for some formulations and not others could unduly bias the model performance. In order to evaluate the influence of these excessive replicates on model accuracy, the program systematically re-ran the prediction process, each time increasing maximum number of experimental replicates per unique configuration and compared the SVM model accuracy (Fig. 4c). There was no change in SVM model performance for predicting particle dispersity when the number of experimental replicates was increased past three and all formulations were restricted to a maximum of n = 6 replicates in the actual models. It is important to note that these models were trained using the default settings (i.e. default hyperparameters) of the *scikit-learn* module, and while hyperparameter tuning to find the best parameters could be performed, it was our intention to construct the classification models with a rapid, “hands off” and out-of-the-box approach.

In order to build the SVM model to predict particle stability, the PdI of the liposomes was evaluated over a 3-Day period and the mean PdI over that time (Supporting Figure S3) was compared to a fixed value of 0.220 via a one-way, one-sided T test. Formulations with a mean PdI significantly greater than 0.220 (*p*-value < 0.05) were classified as unstable. Classification algorithms were then trained on these *p*-values to build a model capable of predicting whether a formulation would be stable or unstable (Supporting Table S2). The SVM model for predicting liposome stability was found to be approximately 92% accurate and had a predicted accuracy of 88 ± 5% with stratified *k*-fold cross-validation. Once the models were trained to predict liposome dispersity or liposome stability, they could be run in a “recall” mode to make predictions over a wide range of theoretical microfluidic parameters. Figure 4d and e show prediction heatmaps marking zones where liposomes are expected to be monodisperse or polydisperse (Fig. 4d), or where they are anticipated to remain stable for at least 3 Days (Fig. 4e). Overlaid on top of the heatmaps are the summary measurements (i.e. filled diamonds) which show the mean results found experimentally. It becomes evident that using a low TFR (< 8 ml/min) is critical for successfully developing viable and stable liposomes with this specific lipid formulation. Figure 4d shows the results of an SVM model using multiple classifications of dispersity (e.g. PdI ≤ 0.200, PdI ≤ 0.300, and polydisperse), while the prediction of stable areas (Fig. 4e) relied on a more strict definition of stability (i.e. PdI ≤ 0.220 over 3-days). A similarly strict interpretation of monodisperse and polydisperse was shown in Supporting Figure S4.

The use of pre-built tools such as the *scikit-learn* module in Python offers an out-of-the-box approach to generate supervised machine learning models to streamline microfluidic-based nanoparticle synthesis. Here the objective was to understand two specific and important questions regarding the microfluidic synthesis of liposomes: (i) could supervised machine learning models or artificial neural networks predict the dispersity of liposome formulations based on the microfluidic synthesis parameters; (ii) could analysis of liposome stability over time be used to predict those formulations which remain stable over a period of days? Certainly, the formation of liposome in a microfluidic, micro-mixing scenario can be described theoretically, and others have reported advanced statistical methods for comparatively deciphering how microfluidic synthesis parameters affect liposome size and stability (Bannigan et al. 2021; Bartheldyová et al. 2018; Kastner et al. 2014; Ocampo et al. 2021; Sedighi et al. 2019; Zook and Vreeland 2010). Here, a specific formulation is described with a fixed lipid ratio of 6:3:1 for DPPC, cholesterol, and DSPE-PEG2000, the presented work provides a point from which a larger database could be built, and the scope of the machine learning algorithms could be expanded to predict a wider array of particles. In fact, such an open-source solution points towards the potential of community-driven machine learning models where large, active databases mean dynamic, evolving machine learning models that can provide a means to rapidly identify formulations that will successfully yield particles.

#### Artificial neural networks for predicting liposome size

Artificial neural networks (ANN) are computational models which use learning algorithms that mimic a simplified brain’s cerebral activity to process and store information. For their capacity to analyze large amounts of data and detect complex patterns, ANN can be effectively applied to a vast array of problems, many of which would be too complex to be solved with theoretical models. As mentioned, in this work the experimental library of different liposomal configurations was exploited to develop neural network models capable to predict the liposome size as a function of the different fabrication parameters. To this aim, the dynamic light scattering measurements were divided in two subsets based on the inclusion of the therapeutic agent: empty liposome (- Curc) and curcumin-loaded liposomes (+ Curc). Consequently, the ANN models were trained only on the remaining three engineering parameters: total flow rate, aqueous:organic volume ratio, and total lipid concentration. Each of the two DLS measurement sets was split into a training data set (85% of observations) and a validation data set (15% of observations), used to monitor ANN overtraining and model integrity. The validation samples were selected on a random basis so that would not be limited to a specific subset of liposomal configurations.

**Fig. 5:**
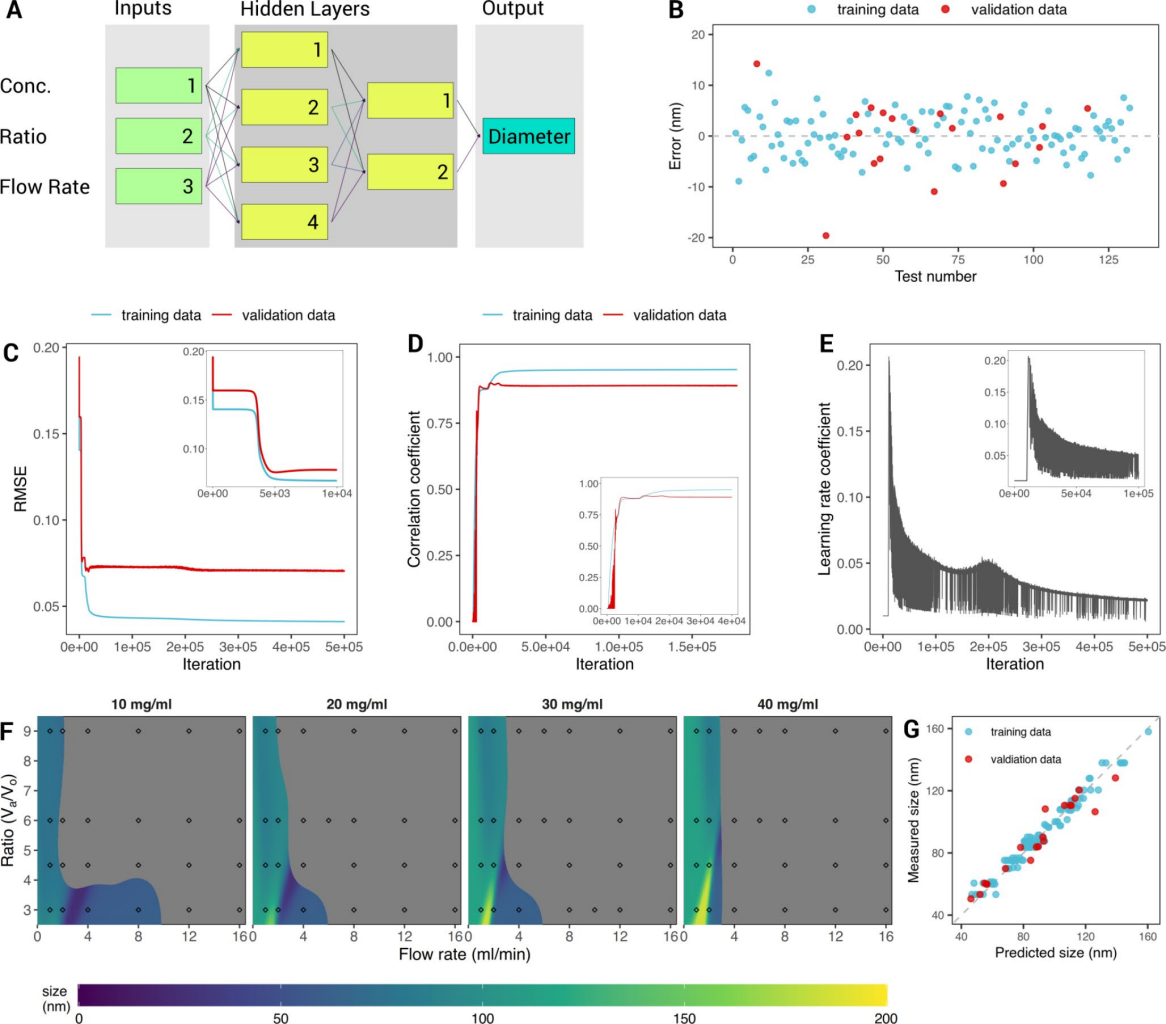
Artificial Neural Networks training to predict the liposome size. (A) Schematic representation of the neural network including three input nodes (green labels); two hidden layers made of 4 and 2 nodes respectively (yellow labels); one output node (cyan label); (**B**) Output error between the predicted and measured particle diameter vs. the training pattern sequence number. Blue dots show the error recorded for the training set, while red dots indicate the error recorded on the validation set. (**C**) Root mean square error (RMSE) of the training and validation sets (blue and red, respectively) vs. iterations. Inset image shows the plateau recorded during the first 10,000 iterations. (**D**) Correlation coefficient of the training (blue) and validation (red) data sets as a function of the iteration number. The optimal agreement has a correlation coefficient equal to 1. Inset image shows the first 40,000 iterations (**E**) learning rate coefficient vs. iterations. (**F**) Prediction heatmaps, showing the calculated diameter for particle synthesis conditions not used for training or validation. The filled diamonds are the mean results of the experimental measurements; the grey areas mark the unstable field (liposomes predicted to be polydisperse, i.e. PdI ≤ 0.220). (**G**) Model predictions vs. the measured particle diameters, the bisector line shows the optimal agreement

Only the experimental data set returning a monodisperse population of liposome were used (PdI ≤ 0.220 on Day 0, immediately after synthesis) to build models for quantitatively predicting the liposome size. As a consequence, the experimental library defined above was significantly reduced, thus requiring a careful design and modulation of the neural networks. ANN models were trained using highly optimized backpropagation training algorithms and the layer-to-layer transfer functions were customized depending on the training characteristics of the first trial neural nets. Fully connected, non-hybrid neural networks, implementing the sigmoid transfer function, proved to be the most suitable. The DLS measurements showed that empty liposomes were significantly more polydisperse compared to curcumin-loaded liposomes (~ 74% compared to 65%, respectively), and exhibit a larger variation in size (Supporting Figure S5). Indeed, a larger number of liposomes returned a PdI < 0.220, especially at lower total lipid concentrations, for the subset of curcumin-loaded liposomes. Nevertheless, for both subsets (+ Curc and – Curc), the same type of ANN model was identified as the most reliable. In Fig. 5a, the optimized network topology is presented: a total of four layers were used to properly model the significant nonlinear behavior of the experimental data. For both liposomal configurations, the ANN model was fed at the input with the three varying synthesis parameters (total flow rate, aqueous:organic volume ratio, total lipid concentration), then accounted for two hidden layers with 4 and 2 neurons, and ended with one-node output layer (the predicted liposome size). The ML model learning statistics revealed that all the microfluidic variables played a significant role on the prediction of the liposome size, with the total fluid rate providing the most relevant contribution (45%), followed by the total lipid concentrations (33%); and, finally, the aqueous:organic volume ratio (22%).

Figure 5b,c shows the difference between the predicted and measured particle size versus the training pattern sequence number and the root mean square error (RMSE) history plot, respectively, for both the training (cyan dots/line) and validation (red dots/line) data sets of the curcumin-loaded liposomes. The graphs for the empty liposomes are similar. The error history of the training data displays the rate of network learning: it plateaued when the learning process reached its maximum level. During the initial phase of the training process, corresponding to the first 20,000 iterations, it showed a few preliminary plateaus in the error level. The first plateau was reached around a few hundreds of iterations returning a RMSE of ~ 0.15 (inset of Fig. 5c), where no significant learning took place, followed by a steep decrease in the training error below 0.05 at ~ 5,000 iterations, where accelerated periods of learning took place (inset of Fig. 5c). After that, the error history showed minor oscillations representing training instabilities, which were damped by the activation of the learning rate control. The validation RMSE history had a similar trend and represented the model quality, depicting the accuracy of the network predictions for cases not used in the training process. Note also that the RMSE vs. iteration plot helps determine how well the ANN generalizes the learned information: a positive slope for RMSE curve would imply overtraining, which is not observed in this case. Continuing with the training of the network after it reached the RMSE minimum in the validation history could affect the predictive capabilities of the model. Over-trained nets usually show limited capacity for the generalization of concepts.

Figure 5d shows the correlation coefficient history curves for the training and validation data sets, which measures how well the network predictions trend with the targets. As expected, these plots should have an opposite behavior as compared to the RMSE: the correlation coefficient increases as the ANN is trained and the corresponding RMSE decreases. Additionally, the correlation coefficient plots provide quantitative information, where a coefficient equal to 1 identifies a perfect linear correlation and 0 is associated to a random correlation. Therefore, the correlation coefficient history plot for the validation data set (Fig. 5d, cyan line) gives a measure of how well the network predictions correspond with measured values for cases outside the training set (i.e. unseen by the ANN), and can give useful indications as to the onset of the overtraining process (a plateau followed by a negative slope). The learning rate coefficient (LRC) versus iteration number is reported in Fig. 5e. The LRC affects the algorithm’s rate of learning within the backpropagation training paradigm. An automatic control routine was inserted to check the coefficient automatically, implementing an effective tool that accelerated the learning process and prevented divergence. It drove LRC higher or lower in a systematic fashion depending on the current learning activity. If at any time the network showed signs of instability (seen as oscillations in the training error), LRC would be lowered to damp the instabilities thus preventing training divergence.

Upon training, the neural network models could be run in ‘recall’ mode to calculate the liposome size over a wide range of microfluidic parameters. Figure 5f presents the prediction heatmaps showing the size of the curcumin-loaded liposomes for different synthesis conditions. Overlaid on top of the heatmaps are the summary experimental measurements (i.e. the filled circles) which show the mean results. The gray areas mark the unstable fields where the SVM model predicted polydisperse liposomes. Finally, Fig. 5g displays a quick overview of the model showing the close correlation between predicted liposome size versus the measured size via the correlation coefficient (R^2^ value). The R^2^ for the entire data set (i.e. training data + validation data) was calculated at 0.959, while the R^2^ for only the training data was 0.958.

Others have previously applied ANN and ML algorithms to construct predictive models for nanoparticle synthesis. Amani et al. (2008) constructed an ANN for predicting the size of nanoemulsions synthesized via a probe sonicator. Input factors such as surfactant percent, ethanol and oil percents, concentration of budosenide (as a model therapeutic), saline normality, input energy, and rate of energy applied were considered in building the ANN. The ANN modeled the formulation with R^2^ values of 0.98 for the training data, 0.92 for the test data (10% of the initial data set that was set aside for validation), and 0.89 for the validation data set (an addition 15 experiments performed to validate the model). Similarly, ANN have been applied for the prediction of size of PLA-PEG-PLA nanoparticles (Asadi et al. 2011), other polymeric nanoparticles (Youshia et al. 2017), and CdSe nanoparticles (Orimoto et al. 2012). Wu et al. (2022) reported the application of ML towards predicting the characteristics of chitosan nanoparticles formulated with a multi-inlet vortex mixer fluidic device and flash precipitation. ML models were then built in python, and took into account various synthesis parameters, namely molecular weight of the cationic polymer, polymer concentration, number of reactive groups in the monomer of the anionic polymer, molecular weight of the anionic polymer, and flow speed. Although not microfluidic synthesis, this study similarly was able to quickly test a number of different ML algorithms to identify the most accurate for predicting diameter and PdI. Thus, it is clear that AI and ML will play an increasingly important role in the development of nanomedicines. The aggregation of these data could be applied towards streamlining the development process.

## Conclusions

Microfluidics offers a scalable approach towards the development of nanomedicine formulations. The application of AI techniques, such as ML, to aid in optimizing nanoparticle synthesis is, in a sense, inevitable. Here, we presented an open-source framework based on the python programming language, which was combined with the development of an ANN to provide a roadmap for the development of therapeutic liposomes via microfluidic self-assembly. The two support-vector machines (SVM) models, built to predict liposome dispersity and stability, were able to reach 93% and 92% accuracy, respectively. Moreover, the ANN could predict curcumin-loaded liposome diameter with a correlation coefficient of 0.927. These data present a potential platform, in which an increasingly growing foundational database can be expanded to obtain powerful, predictive AI models for optimizing nanomedicine development.

## Materials and methods

**Materials.** 1,2-distearoyl-sn-glycero-3-phosphoethanolamine-N-[succinyl(polyethylene glycol)-2000] (DSPE-PEG-COOH), 1,2-Dipalmitoyl-sn-glycero-3-phosphocholine (DPPC), cholesterol were purchased from Sigma-Aldrich (SaintLouis, Missouri, USA). Curcumin (CURC) was obtained from Alfa Aesar (Haverhill, Massachusetts, USA). All reagents and solvents were used without further purification.

### Synthesis of liposomes

Liposomes were prepared using the NanoAssemblr® Benchtop from Precision NanoSystems Inc. (Vancouver, BC, Canada). DPPC, cholesterol, DSPE-PEG2000 (6:3:1) with and without curcumin (50 μg for each sample) was dissolved in EtOH. The organic solvent and an aqueous solution of PBS (pH = 7.4, 1X) was injected into the two inlets of the NanoAssemblr®. Aqueous dispersions of the liposomes were collected from the outlet, resulting from the mixing of two adjacent streams and dialyzed against PBS a 4 °C in order to remove the excess EtOH. For this study, the TFR, the concentration of organic phase (mg/mL) and aqueous:organic volume ratio were changed. Specifically, the TFR used was from 1 to 16 mL/min, the organic concentrations were 10, 20, 30 and 40 mg/mL while the aqueous:organic ratios used were 3, 4.5, 6 and 9 for empty and loaded nanoparticles.

### Characterization of liposomes

Particles size, polidispersity index (PdI) and ζ-potential were measured using dynamic light scattering (DLS, Malvern Zetasizer Nano S). All formulations were maintained in physiologic temperature (37 ± 2 °C) under agitation. At specific time points (1, 2 and 3 days) samples were taken and their physical features were examined.

### Supervised machine learning to determine particle dispersity and stability

A supervised machine learning model was constructed using the open-source Python module *scikit-learn* (Pedregosa et al. 2011). Data from the DLS experiments were randomly divided into training and validation data sets, with 85% of the data being used for training and 15% being used for validation. Within both of these data sets, observations (i.e. the individual DLS measurements) were classified as either monodisperse (PdI ≤ 0.220) or polydisperse (PdI > 0.220). Furthermore, a different classification scheme was tested where formulations were classified as “PdI ≤ 0.200”, “PdI ≤ 0.300” or “polydisperse” (i.e. PdI > 0.300). The features used to predict particle dispersity were the particle synthesis parameters (i.e. TFR, aqueous:organic ratio, organic phase concentration, and curcumin loading).

The features of the training data set were subset into a matrix *X* and trained on the list *Y*, which were the different known outputs (i.e. dispersity or stability). In order to evaluate particle stability over time, the different observations were grouped by TFR, aqueous:organic ratio, organic phase concentration, and curcumin loading. Then, a one-sided t-test was performed on the grouped observations to determine whether the mean PdI over the three days was greater than 0.220 for each formulation, and a p-value > 0.05 indicated that a particle was stable over the duration of the measurements.

Different classification machine learning models, part of the *scikit-learn* module suite, were used to predict particle dispersity and stability: logistic regression (LOR), linear discriminant analysis (LDA), K-nearest neighbor (KNN), decision tree classifier (CART), Gaussian Naive Bayes (GNB), and support vector machines (SVM). For training the classification model to predict dispersity, only the so-called “Day 0” observations were used, i.e. the measurements made directly after synthesis was completed. A stratified *k*-fold cross-validation (k = 10) was run to predict the accuracy of each classification model. The actual accuracy of the model was estimated by checking the different models on the validation data set, and comparing the predicted output with the actual measured values. Ultimately, the SVM was selected to predict particle dispersity and stability.

A program was written to investigate the effect of data set size, number of unique formulations, and the number of experimental replicates per formulation on SVM model accuracy. First, only the measurements taken on “day 0” were subset, and then measurements were randomly removed to provide a data set of sizes *n* = 40 to 779 (the full data set) measurements. Then the holdout with random resampling method was run to evaluate the accuracy of the SVM model. Simultaneously, the program identified the number of unique formulations in each data set of size *n*. Because there were many, but also uneven numbers of replicates run for the different formulations (ranging from 3 to 15 replicates per formulation), the program also systematically restricted the number of replicates from 3 to 15 for each formulation in order to evaluate the effect on SVM model accuracy.

### Artificial neural network to predict liposome size

Data from the DLS experiments showed that empty liposomes are more unstable (PdI > 0.220) and return a wider size variation across multiple measurements as compared to curcumin-loaded liposome. For instance, for a total lipid concentration of 10 mg, an aqueous:organic phase volume ratio of 1:3, a total flow rate of 2 ml/min, the resulting liposome have diameters ranging from ~ 150 up to 300 nm. A slightly higher number of liposomal configurations with PdI < 0.220 was observed for curcumin-loaded liposomes, especially for the 10 and 20 mg total lipid concentrations. Only stable configurations were considered to build a model for predicting the liposome size, thus leading to a significantly smaller DLS measurement data set. Incidentally, this required a more careful selection and design of the neural network models.

To that aim, a customized software was developed, based on feedforward neural networks and backpropagation paradigm, which is an extremely effective machine learning algorithm, suitably exploited for training ANN. The software allows choosing the net topology, including the number of hidden layers and the number of neurons per layer, the connection design, the node-to-node transfer function, the training parameters and the split of the learning database into a training set and a validation set. A continuous monitoring of the trend of the root-mean-square error (RMSE) of the training and validation set was included to check for overtraining and model integrity analysis. The convergence of the training process was improved by means of an automatic control of the Learn Rate Coefficient (LRC) and the momentum factor (MF). The code used a sophisticated control scheme to adjust the LRC and MF to keep network training progressing at a near optimal pace while avoiding instabilities and promoting rapid learning. Moreover, a routine for a systematic interrogation of the network characteristic was implemented to analyze the hidden node contributions to the model performance. This routine allowed one to identify the minimum necessary number of neural units in each hidden layer, thus minimizing the complexity of the model topology. This feature turned out to be particularly useful, given the relatively small subset of DLS measurements for the training and validation data sets. The ML neural model was designed to have relative strengths of all the output connections for each layer in the range of 15–52%.

### Electronic supplementary material

Below is the link to the electronic supplementary material.

Supplementary Material 1

## Data Availability

The data supporting the findings of this study are available within the paper and its Supplementary Information files. Should any raw data files be needed in another format they are available from the corresponding author upon reasonable request.
