# Supplementary material for: Machine learning instructed microfluidic synthesis of curcumin-loaded liposomes
Source: Biomed Microdevices. 2023 Aug 5;25(3):29. doi: 10.1007/s10544-023-00671-1 (PMC10404166; doi:10.1007/s10544-023-00671-1)
Supplement: Supplementary file 1 — Supplementary Material 1 [file 10544_2023_671_MOESM1_ESM.docx]

# Supporting Information

**Machine Learning Instructed Microfluidic Synthesis of Curcumin-loaded Liposomes**

Valentina Di Francesco^1†^, Daniela P. Boso^2†^*, Thomas L. Moore^1†^,

Bernhard A. Schrefler^2,3‡^, Paolo Decuzzi^1‡^*

*^1^Laboratory of Nanotechnology for Precision Medicine, Istituto Italiano Di Tecnologia, Via Morego 30, 16163, Genova, Italy*

*^2^Department of Civil, Environmental and Architectural Engineering, University of Padova, Via Marzolo 9, 35131, Padova, Italy*

*^3^Institute for Advanced Studies, Technical University of Munich, Lichtenbergstraße 2 a, 85748, Garching, Germany*

^†^ D.P. Boso, V. Di Francesco and T.L. Moore contributed equally to this work

^‡^ B.A. Schrefler and P. Decuzzi share the senior authorship for this work

Corresponding author: Daniela P. Boso, PhD – [daniela.boso@dicea.unipd.it](mailto:daniela.boso@dicea.unipd.it);

Paolo Decuzzi, PhD – [paolo.decuzzi@iit.it](mailto:paolo.decuzzi@iit.it)

**Supporting Figures**


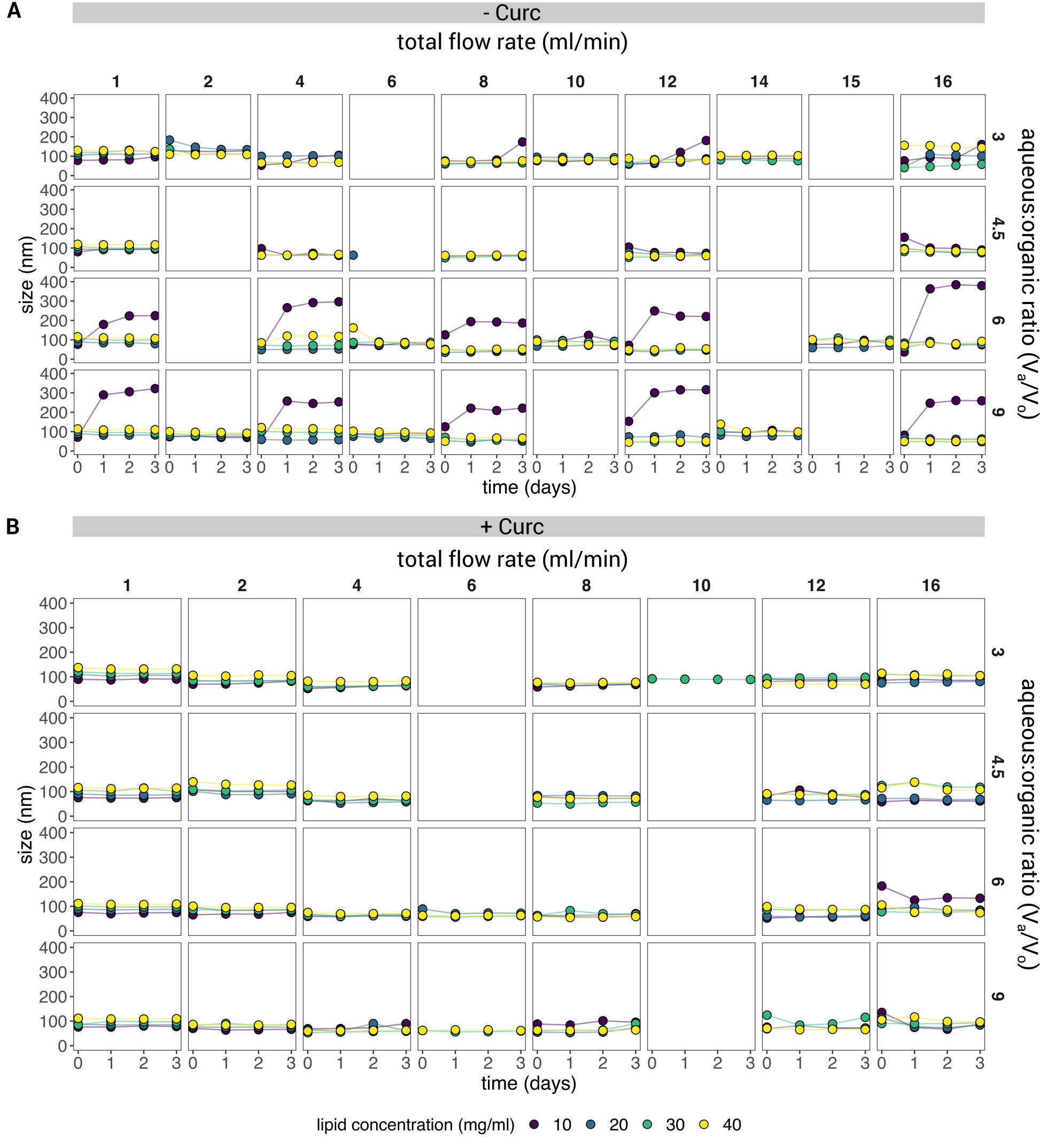


**Supporting Figure S1.** **Characterization of the liposome hydrodynamic diameter over time.** Formulations are split by the empty liposomes (- Curc) and those loaded with curcumin (+ Curc), total flow rate (TFR) as well as the aqueous to organic flow rate ratio (FRR). Particles were characterized over three days with dynamic light scattering (DLS).


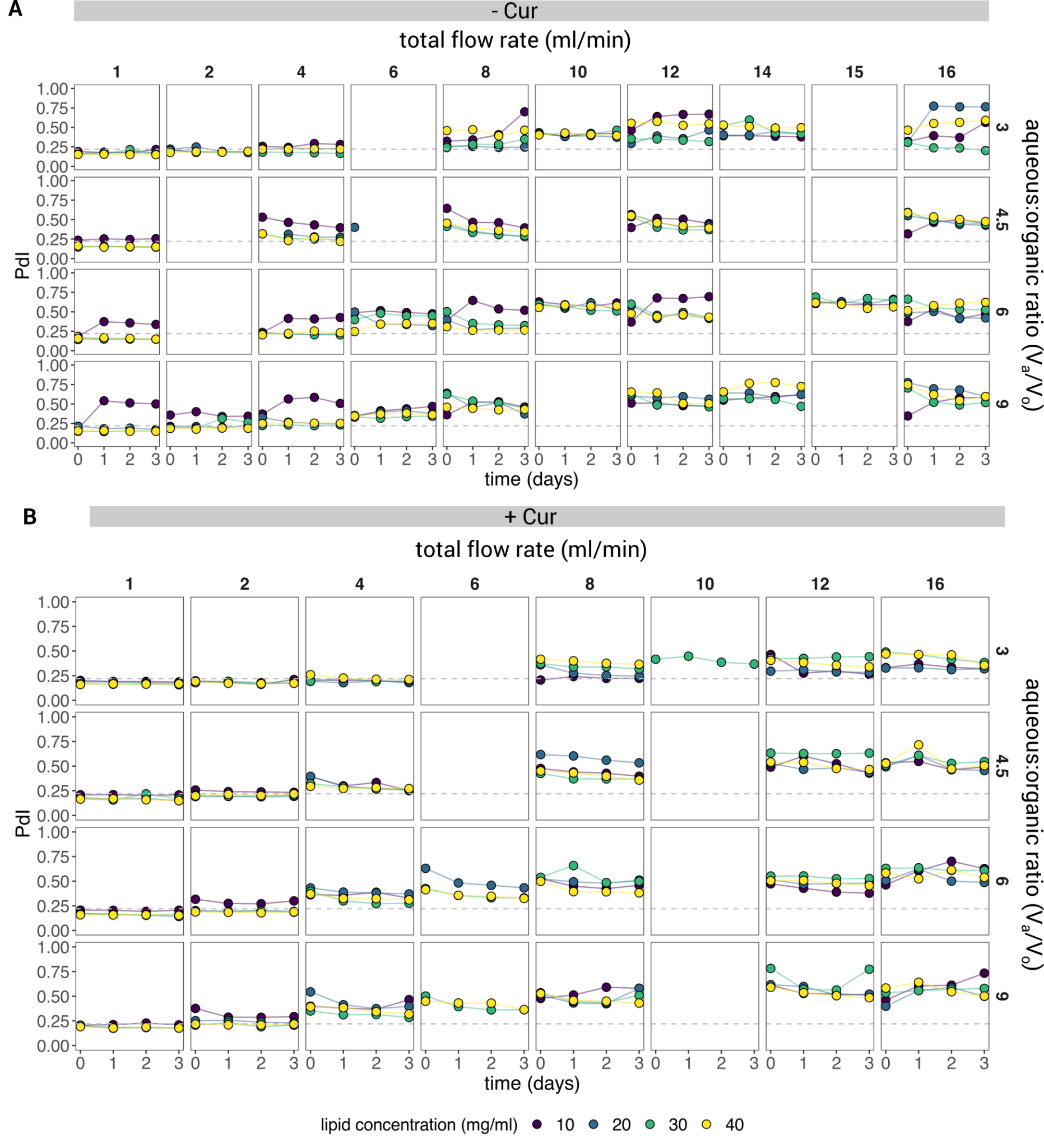


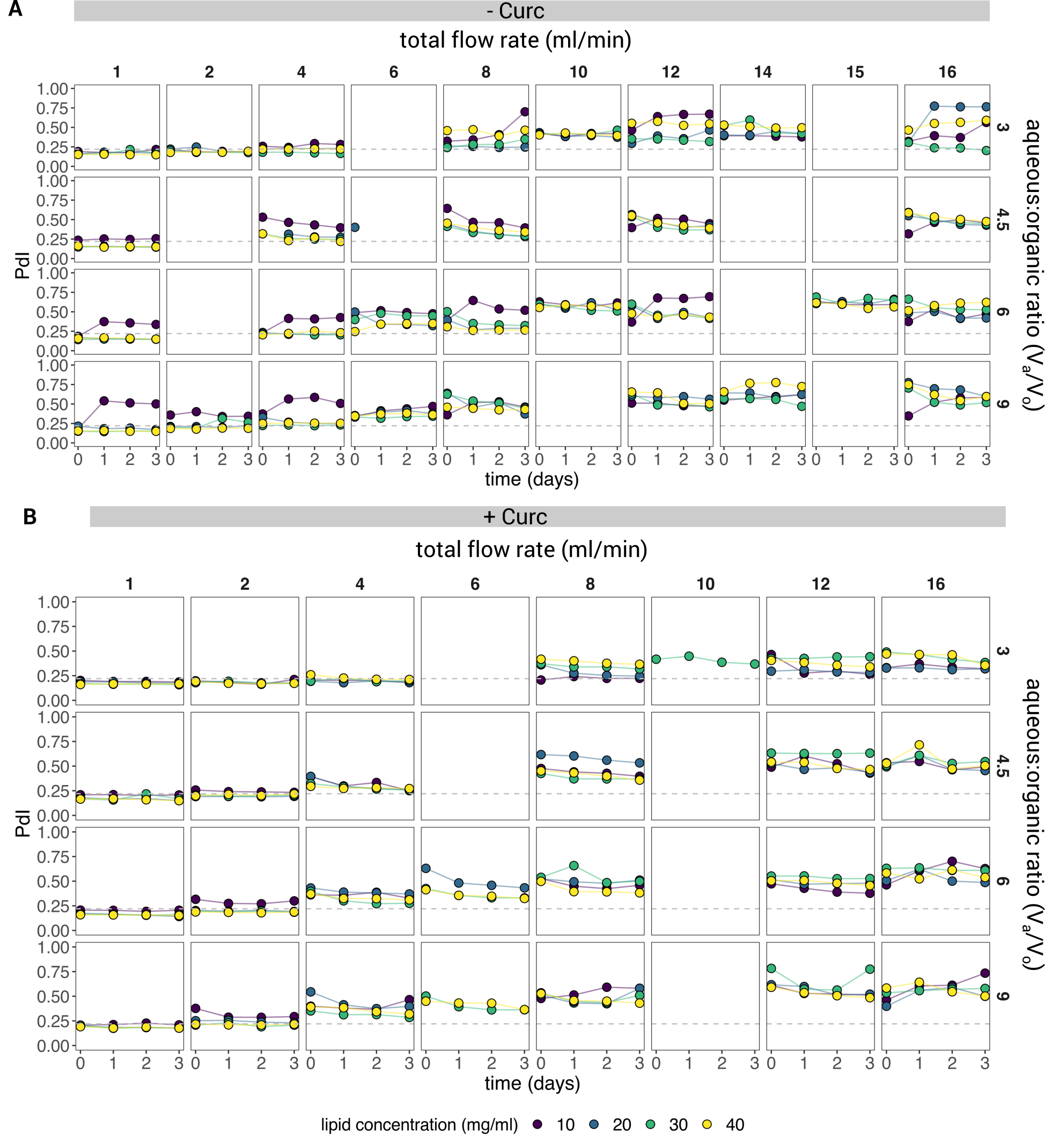
**Supporting Figure S2.** **Characterization of the liposome polydispersity index (PdI) over time.** Formulations are split by the empty liposomes (- Curc) and those loaded with curcumin (+ Curc), total flow rate (TFR) as well as the aqueous to organic volume ratio. Particles were characterized over three days with dynamic light scattering (DLS).

|  |  |
| --- | --- |
| **Total number of measurements**  *(including all the different formulations and all the technical replicates)* | **3518** |
| Total number of measurements (with n ≤ 6) | 1311 |
| **Number of unique formulations [Day 0]**  *(including the different formulations only)* | **218** |
| Total number of measurements [Day 0, n ≤ 6] | 779 |
| **Number of unique monodisperse formulations [Day 0]** | **72** |
| Total number of monodisperse measurements [Day 0, n ≤ 6] | 236 |

**Supporting Table S1.** Summary table for the number of liposome formulations and DLS measurements comprising the total data. (n = maximum number of technical replicates considered for each unique formulation)


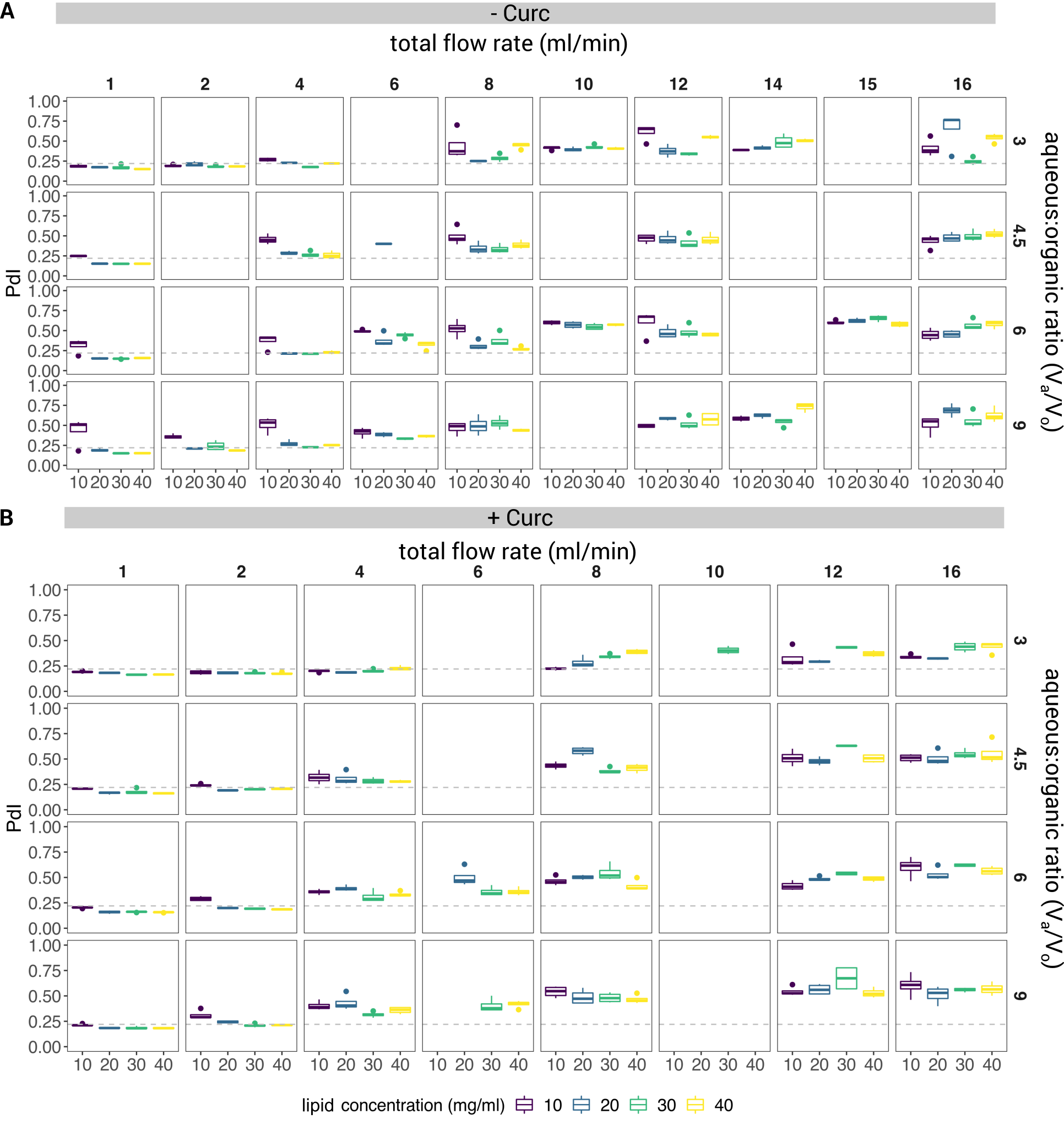

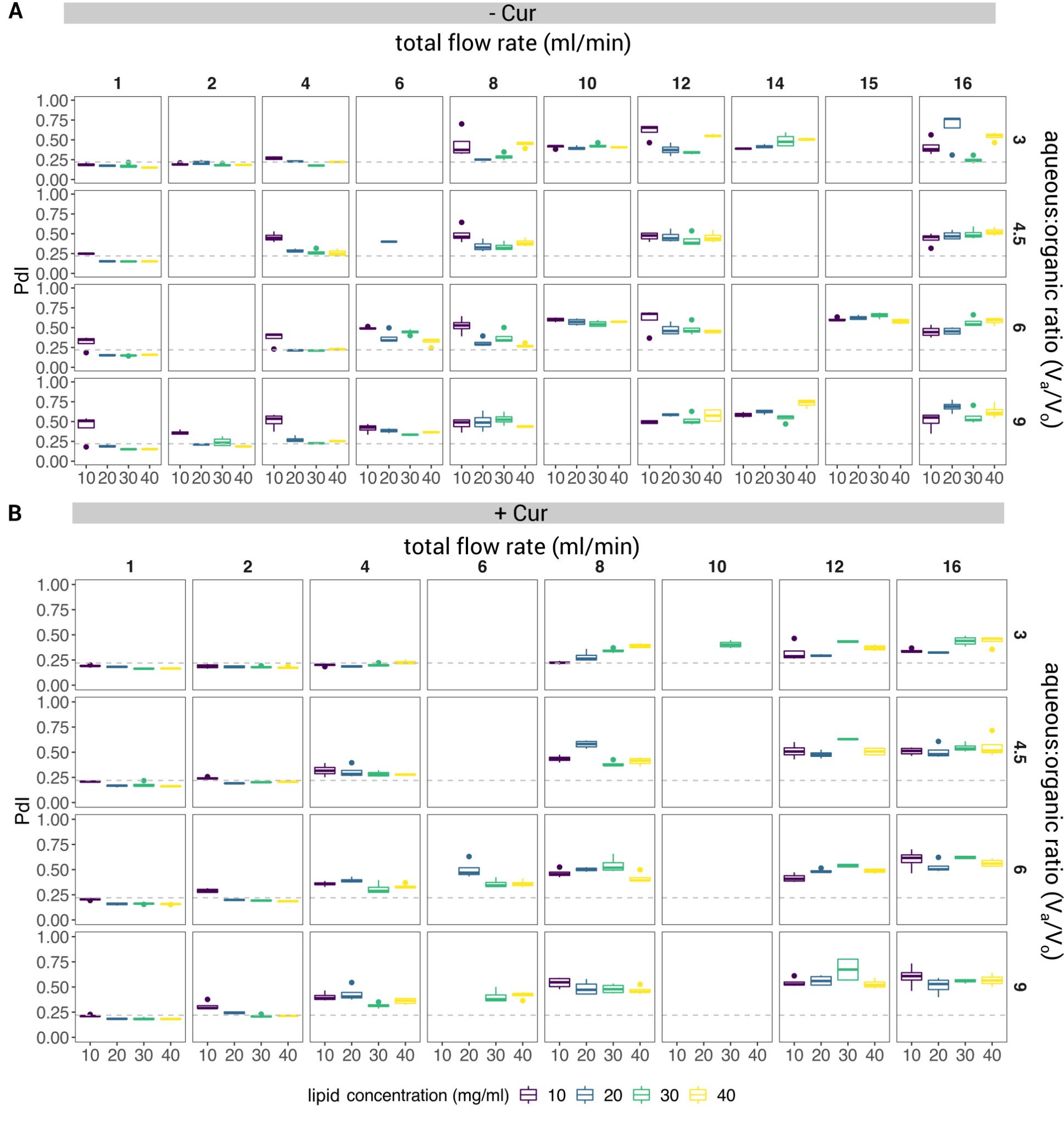
**Supporting Figure S3.** **Summary of liposome PdI measurements over time.** All measurement timepoints were grouped and the aggregate PdI was compared to a reference PdI of 0.220 in order to determine whether liposomes remained stable over the 72 hr period. Formulations are split by **(A)** the empty liposomes (- Curc) and **(B)** those loaded with curcumin (+ Curc), total flow rate, the aqueous to organic volume ratio, as well as total lipid concentration.


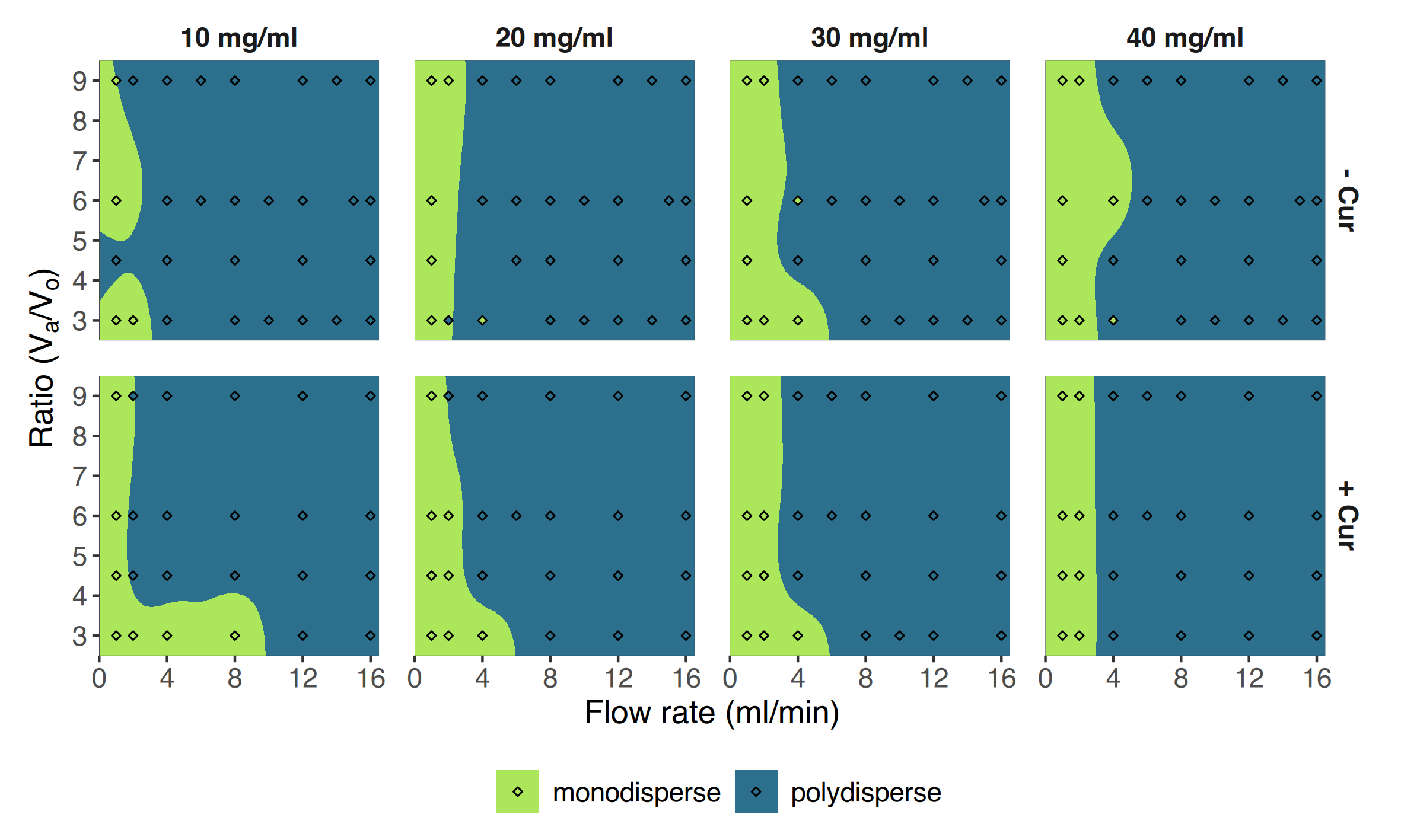


**Supporting Figure S4. Prediction heatmap for a support vector machines (SVM) model trained to predict dispersity.** Liposome formulations were rigidly defined as monodisperse (PdI ≤ 0.220) or polydispserse (PdI > 0.220). Heatmaps show the predicted formulation, and filled diamonds show the actual experimental outcome.


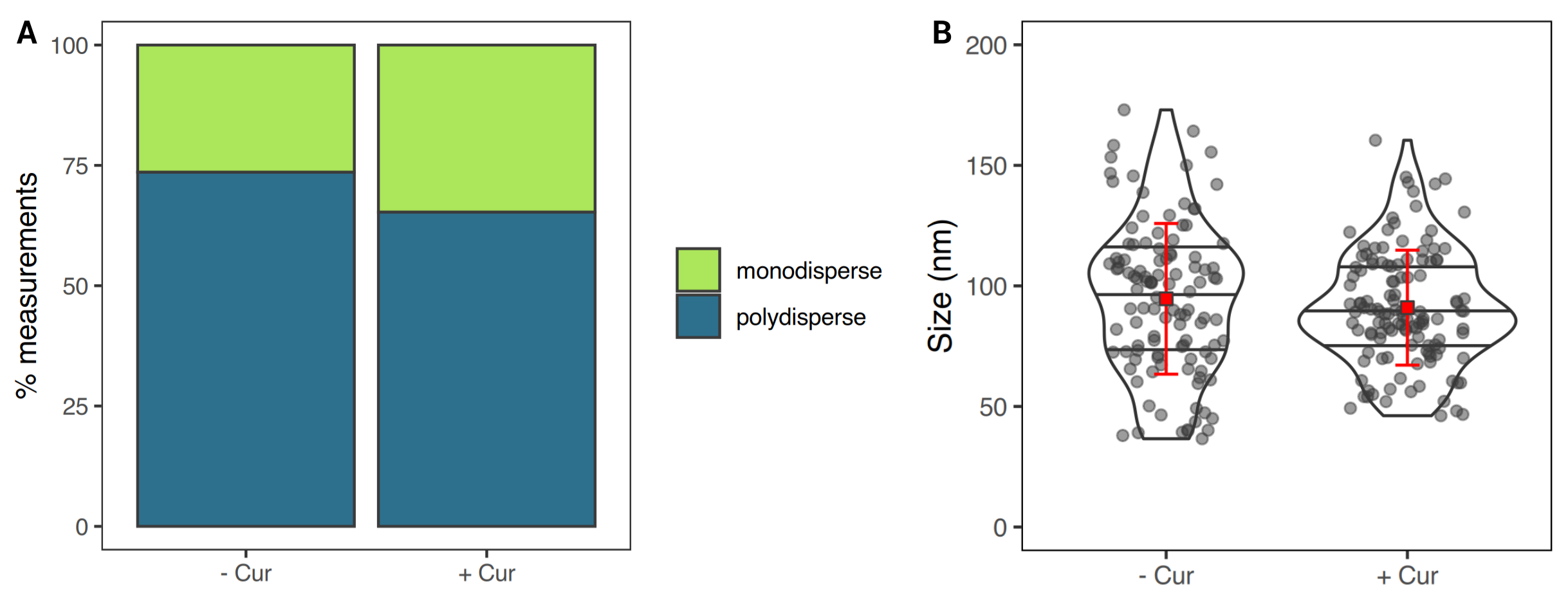


**Supporting Figure S5.** **Summary plots of liposome dispersity and size distributions.** **(A)** Summary plot of liposomes as monodisperse (PdI ≤ 0.220) or polydisperse (PdI > 0.220) based on whether the formulation was “empty” (- Cur) or included curcumin (+ Cur). The total number (%) of monodisperse and polydisperse formulations between – Cur and + Cur was shown to be significantly different (*p*-value = 0.016) by a Pearson’s Chi-squared test. **(B)** The distribution of liposome sizes based on curcumin-loading across all microfluidic conditions was shown to be greater for – Cur formulations compared to + Cur fomulations. Violin plots show 25, 50, and 75% quantiles and red squares show mean ± std. dev.

| **Classification model** | **Predicted accuracy from**  **stratified k-fold cross-validation**  **(mean % ± SD)** | **Measured accuracy**  **(%)** |
| --- | --- | --- |
| **LOR** | 88.0 ± 3.8 | 91.3 |
| **LDA** | 87.1 ± 4.5 | 89.7 |
| **KNN** | 88.0 ± 4.9 | 92.9 |
| **CART** | 89.4 ± 4.4 | 90.5 |
| **GNB** | 85.5 ± 4.0 | 89.7 |
| **SVM** | 89.0 ± 4.5 | 92.1 |

LOR: logistic regression, LDA: linear discriminant analysis, KNN: k-nearest neighbor, CART: classification and regression trees, GNB: Gaussian Naive Bayes, SVM: support vector machines.

**Supporting Table S3.** Predicted and measured accuracy of various classification models for predicting particle stability (stable or unstable).
